# Supplementary material for: Modulation of Prdm9-controlled meiotic chromosome asynapsis overrides hybrid sterility in mice
Source: eLife. 2018 Mar 14;7:e34282. doi: 10.7554/eLife.34282 (PMC5902161; doi:10.7554/eLife.34282)
Supplement: Transparent reporting form [file elife-34282-transrepform.docx]

***eLife’s* transparent reporting form**

We encourage authors to provide detailed information *within their submission* to facilitate the interpretation and replication of experiments. Authors can upload supporting documentation to indicate the use of appropriate reporting guidelines for health-related research (see [EQUATOR Network](http://www.equator-network.org/%20)), life science research (see the [BioSharing Information Resource](https://biosharing.org/)), or the [ARRIVE guidelines](http://www.plosbiology.org/article/info:doi/10.1371/journal.pbio.1000412) for reporting work involving animal research. Where applicable, authors should refer to any relevant reporting standards documents in this form.

If you have any questions, please consult our Journal Policies and/or contact us: [editorial@elifesciences.org](mailto:editorial@elifesciences.org).

**Sample-size estimation**

- You should state whether an appropriate sample size was computed when the study was being designed
- You should state the statistical method of sample size computation and any required assumptions
- If no explicit power analysis was used, you should describe how you decided what sample (replicate) size (number) to use

Please outline where this information can be found within the submission (e.g., sections or figure legends), or explain why this information doesn’t apply to your submission:

Based on our previous studies, the asynapsis rate was measured by evaluating 100 cells per animal. Briefly, this gives a statistical power ≈ 0.85 (for type I error < 0.05) for detecting a ratio of true asynapsis rates 5% and 15% when each is measured in two animals (simulation using GLMM models).

To determine the relationship between the length of PWD/PWD and asynapsis rate in 2-chr crosses and 4-chr cross, we aimed to have at least two animals for each of the lengths 0 Mb, ~10 Mb, ~20 Mb, ~30 Mb,~40 Mb, and >50 Mb of PWD/PWD on each investigated chromosome.

**Replicates**

- You should report how often each experiment was performed
- You should include a definition of biological versus technical replication
- The data obtained should be provided and sufficient information should be provided to indicate the number of independent biological and/or technical replicates
- If you encountered any outliers, you should describe how these were handled
- Criteria for exclusion/inclusion of data should be clearly stated
- High-throughput sequence data should be uploaded before submission, with a private link for reviewers provided (these are available from both GEO and ArrayExpress)

Please outline where this information can be found within the submission (e.g., sections or figure legends), or explain why this information doesn’t apply to your submission:

We performed F1 cross, three 2-chr crosses, and 4-chr cross using the number of animals and number of cells per animal given in Table supplements 1, 3-8, and 12.

For the asynapsis rate, there were biological replicates of ~100 cells per animal.

For F1 crosses, there are two biological replicates at the level of individual animals.

For 2-chr and 4-chr crosses, there are no biological replicates at the level of individual animals, because the recombination is a random process and our aim was to prepare animals with diverse lengths of PWD/PWD segments.

For RNA FISH and HORMAD2 immunostaining, three animals were used; for each of them 10 images of pachytene nuclei were acquired by confocal microscopy, i.e., 3x10 hierarchical biological replicates.

No technical replicates were used in this study.

For modeling the dependence between the asynapsis rate and the length of the PWD/PWD segment, the segmented regression model was used. For a robust estimation of the change point, the data were pooled from all 2-chr crosses.

**Statistical reporting**

- Statistical analysis methods should be described and justified
- Raw data should be presented in figures whenever informative to do so (typically when N per group is less than 10)
- For each experiment, you should identify the statistical tests used, exact values of N, definitions of center, methods of multiple test correction, and dispersion and precision measures (e.g., mean, median, SD, SEM, confidence intervals; and, for the major substantive results, a measure of effect size (e.g., Pearson's r, Cohen's d)
- Report exact p-values wherever possible alongside the summary statistics and 95% confidence intervals. These should be reported for all key questions and not only when the p-value is less than 0.05.

Please outline where this information can be found within the submission (e.g., sections or figure legends), or explain why this information doesn’t apply to your submission:

Statistical analysis methods are described and justified. Statistical tests are identified for each experiment in the “Results” and “Discussion” chapters. Exact p-values are always reported. Further details are stated in “Supplementary Materials and Methods” chapter “Statistics”. Sample size N, mean, SD, confidence intervals are provided in the Table supplements 1, 3-8, 12.

(For large datasets, or papers with a very large number of statistical tests, you may upload a single table file with tests, Ns, etc., with reference to sections in the manuscript.)

**Group allocation**

- Indicate how samples were allocated into experimental groups (in the case of clinical studies, please specify allocation to treatment method); if randomization was used, please also state if restricted randomization was applied
- Indicate if masking was used during group allocation, data collection and/or data analysis

Please outline where this information can be found within the submission (e.g., sections or figure legends), or explain why this information doesn’t apply to your submission:

Group allocation was not relevant in this study.

**Additional data files (“source data”)**

- We encourage you to upload relevant additional data files, such as numerical data that are represented as a graph in a figure, or as a summary table
- Where provided, these should be in the most useful format, and they can be uploaded as “Source data” files linked to a main figure or table
- Include model definition files including the full list of parameters used
- Include code used for data analysis (e.g., R, MatLab)
- Avoid stating that data files are “available upon request”

Please indicate the figures or tables for which source data files have been provided:

The R code for the simulation used for a comparison of probabilistic distribution of number of symmetric DSBs and probability of proper meiotic synapsis is included.
